# An attempt to identify the issues underlying the lack of consistent conceptualisations in the field of student mathematics-related beliefs

**DOI:** 10.1371/journal.pone.0224696

**Published:** 2019-11-06

**Authors:** José Manuel Diego-Mantecón, Teresa F. Blanco, José M. Chamoso, María José Cáceres

**Affiliations:** 1 Faculty of Science, University of Cantabria, Santander, Spain; 2 Didácticas Aplicadas, University of Santiago de Compostela, Santiago de Compostela, Spain; 3 Faculty of Education, University of Salamanca, Salamanca, Spain; University of Westminster, UNITED KINGDOM

## Abstract

This paper aims to clarify the inconsistencies present in the field of student mathematics-related beliefs. Despite the general agreement about the important role that beliefs play in the learning of mathematics, the study of student mathematics-related beliefs has resulted in a body of uncoordinated research. The lack of consensus on defining and classifying beliefs has generated much confusing terminology, preventing a consistent conceptualization of the phenomenon. To identify the problem underlying existing inconsistencies, we have undertaken a systematic review of the literature to analyse the belief conceptualisations proposed by the most cited authors in this field of research. Our analysis suggests that authors often fail to conceptualise beliefs in four important ways: existing theories related to the phenomenon under research are normally not considered; definitions are often too broad and do not clearly confine the construct under evaluation; and existing beliefs sub-constructs are rarely defined and thus not explicitly distinguished. Our study has also revealed that some of the scales developed to measure the belief constructs lack of content and internal validity. We believe that these findings open new lines of research that may help to clarify the field of student mathematics-related beliefs.

## 1. Introduction

This paper seeks to shed light on the inconsistencies present in the field of student mathematics-related beliefs. A number of influential researchers have undertaken rather thorough reviews to describe mathematics-related beliefs, in general, as an uncoordinated body of research (e.g. [1], [2], [3], [4], [5]). These reviews have mainly focused on identifying contradictory definitions on beliefs, but have rarely analysed the issues underlying such unsystematic research. For instance, Pajares [1], in his well-known paper on teacher beliefs, restricts himself to challenging a number of belief definitions from the existing literature. He highlights the need for an agreed definition to enable more efficient research, but does not explore the reasons underlying the lack of coordination itself. Similar criticism can be applied to Furinghetti’s and Pehkonen’s [4] study of student beliefs. They list a large number of belief definitions from a range of well-known mathematics educators, and examine differences and similarities to conclude that one agreed definition might be unfeasible.

In an effort to bring clarity to the field of student mathematics-related beliefs, some researchers (e.g. [6], [7], [8], [9]) have developed models to classify the existing categories of beliefs. Despite this systematic research, these models have done no more than increasing the complexity of the field as they often propose different categorisations. As indicated by Op’t Eynde [10], and discussed below, some models are even contradictory, merging or separating the same belief categories according to the authors’ interpretation. Different labels are also used with the same belief concept, or the same belief concept is described as including different sub-categories across different models. These contradictions create great confusion when interpreting results and prevent the field from moving forward. Much recent studies ([11], [12], [13]) have aimed to clarify the field by conceptualising affect at a more general level and by proposing future directions, but the confusion still persists.

The aim of the present study is not only to show the existing inconsistencies in the field of student mathematics-related beliefs, as others have earlier done (e.g. [10], [4], [13]), but also to identify the issues underlying such inconsistencies. To achieve this aim we have analysed beliefs’ conceptualisations from those papers most widely quoted in the field, and which we think lead the research area. The feasibility of the conceptualisations was analysed under De Vellis’ psychometrics framework [14]. This framework focuses on conceptualising and measuring elusive phenomena, like beliefs, and suggests that conceptualisations should be undertaken under four main approaches: (1) the phenomenon under research should be well-grounded in the substantive theories; (2) the specificity at which it is intended to be assessed should be clearly stated; (3) their sub-dimensions should be explicitly defined; and (4) consistent and valid scales should be provided to adequately measure each sub-dimension. Our analysis shows that authors in mathematics education typically fail to effectively address these four steps, being recognised by De Vellis [14] and Marsh [15] as essential for establishing consistent conceptualisations. As we hope to show in the following sections, these failures generate a large body of unsystematic research and contradictory results, compounding the inconsistent conceptualisations.

The paper is presented in four sections: firstly, the main inconsistencies in defining and categorising beliefs in the field of mathematics education are identified; secondly, the methodology used to detect the issues underlying such inconsistencies is described; thirdly, the results gained from the analysis are presented; finally, the paper concludes by highlighting the main points of the study and the implications for devising a framework that helps to clarify the field.

## 2. Literature review

Although in mainstream psychology and related disciplines there is also some disagreement about how to define beliefs, authors tend to agree about some aspects of this phenomenon. In general beliefs are considered to be socially constructed, specific to the individual, and thus subjective. An individual continuously receives perceptions from the world around him/her and compares his/her beliefs with new experiences and other beliefs they hold [16]. This continuous evaluation and change means that any new belief is automatically incorporated into an existing belief structure. Although constructed from subjective perceptions, beliefs (as well as desires) are stored in the brain, and can be retrieved from memory [17]. In other words, they are mental states which depend on perceptions and not direct copies of the environment ([17], [18]). Since beliefs are traditionally treated as the simplest form of mental representation and therefore one of the building blocks of conscious thought [19], it is accepted in psychology that ‘beliefs’ are represented in the mind as sentence-like constructs ([20], [17], [21], [22]).

Despite the aforementioned concordances on the nature and functioning of beliefs, researchers on mathematics education have encountered many difficulties for conceptualising beliefs and agreeing a definition. The following reviews the literature on mathematics-related beliefs and shows that the field has turned into a body of uncoordinated research. In particular, definitions (§2.1) and categorisations (§2.2) of beliefs are examined to highlight existing inconsistencies.

### 2.1 Inconsistent definitions of beliefs in mathematics education

While the term ‘belief’ is widely used in the mathematics education literature, its meaning remains contested. Indeed, researchers often construct definitions which contradict those of others. Definitions tend to be construed as either static or dynamic [4]. The former includes statements such as ‘beliefs are’, ‘beliefs constitute’ (e.g. “beliefs constitute the individual subjective knowledge about self, mathematics, problem-solving, and the topics dealt with in problem statements” [23], p.77). In contrast, other definitions stress the dynamic nature of beliefs, that is, how beliefs function (e.g. “beliefs are understandings and feelings that shape the way an individual conceptualises and engages in mathematical behaviour” [24]). Other researchers define beliefs by relating them to knowledge, but there is still no consensus. For example, Pajares [1] and Da Ponte [25] state that ‘beliefs’ and ‘conceptions’ are regarded as part of knowledge, while Andrews and Hatch [26], and Thompson [2] consider both ‘knowledge’ and ‘beliefs’ as components of ‘conceptions’. According to Pajares [1], the main difficulty in defining beliefs is the inability to distinguish ‘knowledge’ from ‘beliefs’. Abelson [27] and Nespor [28] suggest that beliefs are non-consensual and consequently disputable, while knowledge is generally verifiable; that is, beliefs are individual constructs, while knowledge is essentially socially constructed [10]. Furinghetti and Pehkonen [4] propose the distinction between objective and subjective knowledge as a means of distinguishing between the formalised and collectively agreed knowledge that is mathematics, and the individually constructed, experiential and tacit knowledge of the individual. Still some recent studies (e.g. [29], [30]) suggest that beliefs relate to affect rather than knowledge. The confusion increases when, as discussed below, terms such as ‘attitudes’, ‘conceptions’ and ‘views’ have been used in ways suggesting that not only may they be synonyms for ‘beliefs’, but also that they may be different constructs. In this respect, Mason’s ([31], p. 347) tongue-in-cheek summary of the situation has the ring of truth. For researchers in the field, the “first stumbling block is to work out what beliefs actually are, and where they fit into an entire alphabet of associated interlinked terms”.

As with beliefs, researchers often construct their own definitions of attitudes. Goldin ([3], p.61), for instance, states that attitudes are “stable predispositions toward ways of feeling in classes of situations, involving a balance of affect and cognition”. Similarly, Aiken [32] suggests that attitudes may be conceptualised as learned predispositions to respond positively or negatively to certain objects, situations, concepts, or persons. Sloman [33] (in [34], p.97), however, understands attitudes as “a collection of beliefs, motives, motive generators, and comparators focused on some individual, object or idea”. Yet others, such as Rokeach ([35], p.116), refer to attitudes simply “as an organization of interrelated beliefs around a common object, with certain aspects of the object being a focus of attention for some persons, and other aspects for other people”.

The main difficulty in defining attitudes is the inability to distinguish ‘beliefs’ from ‘attitudes’. Several authors do not accept the possibility of isolating these concepts, and thus define attitudes as collections of beliefs (e.g. [35], [36]) or consider beliefs to be part of attitudes (e.g. [37], [38] both in [39]). McLeod [7], in his reconceptualisation of the affective domain, has suggested two ways of differentiating these two concepts. The first way concerns the level of intensity of the affects that they describe, increasing in intensity from ‘cold’ beliefs about mathematics to ‘cool’ attitudes related to liking or disliking mathematics. The second way relates to the degree of cognition and the time taken for development; beliefs are more stable than attitudes and have a stronger cognitive component. Despite these differentiations, new definitions of beliefs and attitudes are continually being created and the confusion persists (see [3]).

Although the term ‘conceptions’ is less used than ‘beliefs’, the literature on ‘conceptions’ is also ambiguous. [2] defines teachers’ conceptions of mathematics as their “conscious or subconscious beliefs, concepts, meanings, rules, mental images, and preferences” (p.132). Da Ponte [25] stresses that conceptions are the underlying organising frames of concepts and are essentially cognitive in nature. In contrast, Ernest [40] suggests that they are belief systems and, in essence, affective. Lloyd and Wilson ([41] in [4]) use the term ‘conceptions’ to refer to a person’s general mental structures that encompass knowledge, beliefs, understandings, preferences and views. Saari ([42] in [8]), who concurs with Furinghetti [43], defines them as conscious beliefs. For her, conceptions are higher-order beliefs, based on a reasoning process for which the premises are conscious. Freire ([44] in [4]) explain conceptions as being a set of ideas, beliefs, understandings and interpretations of pedagogical practices concerning the nature and content of a discipline.

However, the difficulty arises here in distinguishing between beliefs, conceptions and knowledge. As shown above, while some researchers consider ‘beliefs’ and ‘conceptions’ to be part of knowledge ([1], [43]), others see both ‘knowledge’ and ‘beliefs’ as part of ‘conceptions’ ([2], [26]). The view of the latter authors and that of Op’t Eynde [45] is that beliefs and knowledge are linked and that one influences the other. Consequently, they describe ‘conceptions’ as comprising both cognition and affect, which some authors argue is more an ‘attitude’ (see previous sub-section).

The term ‘views’ is less used in the literature, but it is also contradictory. ‘Views’ is a variation of the concept ‘mathematics world view’ which was originally introduced by Schoenfeld [24]. He used this concept to refer to the individual’s belief structures, or belief systems. Later the term ‘view’ was adapted by others (e.g. [46], [47]), who recognise pupils’ view of mathematics as a wide spectrum of beliefs (and conceptions) which contain, among others, four main components: beliefs about mathematics, the self, mathematics teaching, and mathematics learning. But ‘views’ has received other definitions. For example, Pehkonen [8] has used this term to distinguish a particular kind of conceptions. He states that views are “very near to conceptions, but they are more spontaneous, and the affective component is more emphasised in them” (p.13). For him the cognitive component is more stressed in conceptions than in views, but less than in beliefs. Another use of ‘views’ is that given by Wedege and Skott [48], who use this term to avoid making the distinction between beliefs, attitudes and conceptions.

The above sub-sections show the conceptualisation of beliefs has become a problem in the field of mathematics education and, although several studies have tried to clarify the situation (e.g. [49], [3], [34]), confusion still persists. Indeed, authors such as Thompson [2] and Törner [49] have even suggested it is just a philosophical problem and that it is not realistic to look for an authoritative definition of beliefs. In an attempt to clarify the field, researchers have categorised beliefs in theoretical models. As the following section shows, inconsistent classifications of beliefs have been proposed.

### 2.2 Inconsistent categorisations of student mathematics-related beliefs

In this section the most relevant beliefs categorisations in the mathematics education literature are compared to highlight existing inconsistencies. For example, an examination of some of the early work in the field highlight’s Frank’s [50] model, which distinguished five belief dimensions: beliefs about (1) the ability to do mathematics; (2) mathematics as a discipline; (3) the origin of mathematical knowledge; (4) solving mathematical problems; and (5) how mathematics is taught and learnt. Underhill [6] classified pupils’ beliefs into four dimensions: beliefs about (1) mathematics as a discipline, (2) mathematics learning, (3) mathematics teaching, and (4) the social context. Frank’s fourth dimension was not included although Underhill considered ‘beliefs about the social context’ (not stressed by Frank). He also split Frank’s fifth dimension into two: ‘mathematics learning’ and ‘mathematics teaching’. Thus they concur on only one of the main dimensions, ‘mathematics as a discipline’. McLeod [7] proposed four dimensions: beliefs about (1) the nature of mathematics (which includes the first two categories of Underhill’s model); (2) the ‘self’ (which is not clearly differentiated in Underhill’s model); (3) mathematics teaching; and (4) the social context. Both researchers, however, consider the same third and fourth belief dimensions. Kloosterman [51] described a different model with just two main dimensions: beliefs about (1) mathematics and (2) learning mathematics. In the second system, he considers several sub-dimensions, such as ‘beliefs about mathematics learning’, which is considered a main dimension in Pehkonen’s [8] model. Finally, Op’t Eynde [10], in an attempt to fuse the above models, developed a model which comprises three main dimensions: beliefs about (1) mathematics education, (2) the ‘self’, and (3) the class context. These dimensions are interrelated and are constituted by smaller, related sub-dimensions. For example; ‘beliefs about mathematics education’ is comprised of three sub-dimensions: beliefs about mathematics, mathematics learning and problem-solving, and mathematics teaching.

From the above examples, it can be seen that several dimensions, for instance those about ‘beliefs about mathematics’, ‘learning’, and ‘teaching’, are classified in various ways. Some researchers consider ‘beliefs about mathematics’ and ‘beliefs about learning’ as a single dimension [7], while others consider ‘beliefs about learning’ and ‘teaching’ as the same dimension [50], and yet others consider the three dimensions as a single system [10]. Further confusion arises when examples of these dimensions are provided; for instance, Op’t Eynde [10] link ‘mathematics learning’ with statements such as *mathematics learning is mainly memorising*. Spangler [52] uses a similar statement, *mathematics is about having a good memory*, to refer to ‘beliefs about mathematics’, and Pehkonen [8] uses the item *good mathematics teaching includes memorising rules* for ‘mathematics teaching’. In categorising beliefs, some overlap may be expected because beliefs do not occur independently. However, problems arise when categorisations become ambiguous and authors use the same item to exemplify different dimensions as above. The revealed discrepancies are corroborated by the fact that none of the above models have empirical support for the discussed dimensions; see for example, De Corte’s [9] and Lazim’s [53] studies which respectively test Underhill’s [6] and Op’t Eynde’s [10] models. Evidence from the abovementioned literature highlights the need for the mathematics education research community to adopt a more consistent framework of student beliefs. The incompatible and even contradictory classifications clearly lead researchers to different interpretations, fomenting more confusing literature and preventing the field from moving forward.

This entire section shows a clear disagreement on defining and categorising student mathematics-related beliefs and demonstrates the need for a more efficient conceptualisation of the field. The research question that arises here is thus: What are the main issues underlying the inconsistent conceptualisations of student mathematics-related beliefs? The identification of these issues should lead to more systematic research and therefore to a clarification of the field.

## 3. Materials and methods

To answer this question, a first broad review of the literature was undertaken poring over more than 189 mathematics-related beliefs papers from databases like Web of Science, Scopus, and Eric. The search was undertaken by entering key words such as ‘beliefs’, ‘conceptions’ and ‘views’ that, as explained earlier, have been historically used interchangeably in mathematics education. In this first review we scrutinised studies from the 80s, when beliefs were first investigated in the USA to help students to become better problem solvers [50]. These studies were basically aiming the identification of maladapted beliefs through classroom observations. We reviewed articles from the 90s, mostly centred on defining and categorising the beliefs identified in previous works in the literature. Journal articles from this millennium, generally focused on measuring and comparing beliefs across factors such as age, sex and cultural locations, were also revised as well as proceedings of the CERME (Congress of the European Society for Research in Mathematics Education) and ICME (International Congress on Mathematical Education) congresses where the mathematics-related beliefs community has been rather active.

From this first review a purposive sample of 72 papers was selected to undertake a more in-depth analysis of the existing beliefs’ conceptualizations. Well-referenced papers were selected according to Google Scholar and Research Gate citation databases. These papers were written by well-known authors—including Pintrich, De Groot, Bandura, Schommer, Thompson, Pehkonen, De Corte, Op’t Eynde, Kloosterman, Gil-Ignacio, Blanco, Philippou, Furinghetti, Leder, Törner, McLeod and Hannula—who we believe have strongly influenced the field of student mathematics-related beliefs. In the following, examples of the selected papers are provided with their Google Scholar (GS) and Research Gate (RG) citation number: Pintrich and De Groot [54] paper shows 9834 GS and 3395 RG citations; Bandura [55] displays 5616 GS and 2300 RG citations; Pintrich [56] shows 5892 GS and 2876 RG citations; Thompson’s [2] has 3617 GS and 1278 RG citations, Schommer [57] contains 2875 GS and 1309 RG citations, etc. (data retrieved October 1^st^, 2019). Most recent papers as De Corte [9], Sumpter [58], Yang [59], Bofah [60], Ren [61], Hannula [5], and Diego-Mantecón [62] were also selected to cover latest research, although they do not obviously count with so many citations. To avoid sampling bias, chapters from well-known books in the field of mathematics-related beliefs, such as Leder [63], Pepin [64] or Hannula [11] were also examined. Book chapters allow better elaboration of theory and more extensive description of methodology than journal articles and conference papers, suffering often from a tight space restriction. To further enhance the validity of our study, we also analysed the belief conceptualisations of large-scale projects (e.g. TIMSS and PISA) that have been refined across the years in different cultural-contexts. In a similar vein, widely used instruments such as Fennema’s and Sherman’s Mathematics Attitude Scales (FSMAS) [65], Kloosterman’s and Stage’s Indiana Mathematics Belief Scales (IMBS) [66], and Op’t Eynde’ and De Corte’s student Mathematics-Related Beliefs Questionnaire (MRBQ) [67] were also revised.

Importantly, some authors often writing on teacher beliefs/conceptions, as for example Thompson, were similarly included in our data sample, because their conceptualisations have highly influenced the field of student beliefs. For instance, Thompson’s [2] definition of conceptions, cited earlier in this paper, has been regularly adopted across the years to assess student beliefs/conceptions—in 2012 alone, it was adopted by Blomqvist [68]; Oladipo [69]; and Lam [70] among others.

To analyse the beliefs’ conceptualisations of the 72 selected papers we used the framework devised by De Vellis [14], and adopted by others in mainstream education as for example Marsh [15]. De Vellis’ framework aims conceptualising and measuring elusive phenomena, as is the case with beliefs. It is based on psychometrics theory which refers to the theory and technique of measurement of educational and psychological constructs such as knowledge, abilities, beliefs, and attitudes, and focuses on the development of theoretical models to measurement ([71], [72]). Conceptualising unobservable variables or constructs (i.e. abstract variables which cannot be directly measured) is a challenging task because there is no tangible criterion against which to calibrate them. It is thus necessary to have clear ideas about the phenomenon to be measured to serve as a guide to develop its conceptualization. To ensure a sound conceptualisation De Vellis [14] suggests at least four main steps: (1) the construct should be well-grounded in the substantive theories; (2) the specificity at which the construct is intended to be assessed should be clearly stated; (3) the possible dimensions of the construct should be clearly defined; and (4) consistent and valid measurement scales should be designed for assessing each conceptualisation. Constructs can be developed in relatively broad or narrow ways with respect to the situations to which they apply. However, the precision to which a conceptualisation is given is crucial because it may capture several meanings and lead to a confused applicability ([13], [14]). For example although the purpose of a conceptualisation may be to cover only one phenomenon, it may be too general or unbounded covering several meanings and thus leading to different interpretations.

In this study, the analysis of the beliefs’ conceptualisations in the 72 selected papers was undertaken under De Vellis’ framework. That is, the consistency of the conceptualisations was tested against the four dimensions above described, assessing their practical application for measuring beliefs. First, each conceptualisation was confronted to the following questions: Is this conceptualisation well-grounded in the substantive theories? Is this conceptualisation defined at a specific level? Are the specific sub-categories of the construct under evaluation clearly defined? After this scrutiny, the applicability of the conceptualisations for measuring beliefs was evaluated by assessing content validity; that is, by assessing the adequacy and representativeness with which the *proposed* belief statements (or multi-item scales) represent the content area conceptualised [73]. We thus assessed how well the items proposed by the authors sampled the construct of interest. Finally, scale validity was assessed when corresponding; that is, we evaluated whether the item pools proposed to measure the constructs were meeting basic measurement principles such as *internal* and *cross-cross-cultural validity* ([74], [75]). *Internal validity* is the extent to which the research design impacts on the research outcomes, and the extent to which respondents are exposed to factors that can affect the information. A way of increasing internal validity, for example, is to ensure the suitability of the items to the target group, by having the item pool revised by teachers or educators who are in contact with the target group and are aware of its vocabulary [76]. *Cross-cultural validity* aims to ensure that the scales measure what they are intended to measure equally across populations ([77], [78]). This approach is crucial for international comparative studies and also for comparing results across different contexts. To achieve complete cross-cultural validity researchers suggest establishing different sorts of scale equivalence, most commonly conceptual equivalence ([74]). Conceptual equivalence examines whether the concepts used in a study have equivalent meaning in different cultures. According to Smith [79], an optimal translation may not produce conceptual equivalence. For example “the concept equality/egalité is understood differently in the United States, English-speaking Canada, and French-speaking Canada” (p.432).

## 4. Results

We found that often authors writing in mathematics education do not address adequately the four key steps reported by De Vellis [14] as essential for ensuring sound conceptualisations. The analysis revealed that many of the assessed conceptualisations fail in at least one of the four De Vellis’ dimensions, thus showing low applicability for assessing beliefs. Empirical studies usually focused on measuring beliefs do not provide a clear definition and do not acknowledge the existing theories related to the constructs under research, obstructing precise conceptualisations. Theoretical studies, focused on defining and categorising beliefs, fail mainly on delimiting the construct under evaluation. Definitions are generally too broad, leading to confusing and ambiguous categorisations. Neither empirical nor theoretical studies tend to provide detailed descriptions of the specific beliefs sub-categories. That is, different beliefs categories are distinguished but not properly defined. Apart from the above, widely used instruments in the literature were often found to comprise measurement items that lack of internal validity and conceptual equivalence. In turn, the analysis showed that these four identified failures (1- the limited use of the existing theories; 2- the use of broad definitions; 3- the lack of description of the beliefs sub-categories; and 4- the lack of internal consistency of well-known instruments in the literature) lead to a poor applicability of the conceptualisations for the measurement of beliefs, and thus to mistaken judgements about the structure and meaning of beliefs. Explicit examples about the four aforementioned failures are provided below.

### 4.1 Using existing theories related to the constructs

An analysis, for example, of the empirical studies of Pehkonen ([8], [80]), Lazim [53], Rösken [46], Hannula [5] suggests that researchers in mathematics education do not draw sufficiently on the substantive theories related to the constructs. Although studies such as those of Mousoulides [81] locate their research within the relevant beliefs-related theories, the majority of them seem to fail in this respect. For instance, Rösken [46], in a study about the dimensions of students’ views of mathematics, developed scales to measure constructs such as ‘confidence’, ‘enjoyment’ and ‘effort’, but did not acknowledge the relevant social-science theories that should be considered before attempting construct conceptualisation [82]. For example, the social cognitive theory of Bandura [55] could have helped the authors to conceptualise their ‘confidence’ construct, which they failed to define. In the same vein, intrinsic motivation theory [83] could have assisted them to put ‘effort’ and ‘enjoyment’ into context within the surrounding constructs. Such things are important because theory helps to delimit constructs, facilitating scale design [14]. For example, the use of self-concept theory [15] would help researchers to understand that items such as *I am not good in maths*, which relates to ‘self-concept’, are inappropriate for tapping into the construct ‘self-competence’.

There are studies which have formulated a framework, but have failed to clearly state its underlying purpose. Indeed, most proposed frameworks appear to be categorised with little theoretical foundation. Consider for instance Pehkonen’s ([8], [80]) framework of students’ views of mathematics, which includes four main dimensions, namely views about: (1) mathematics, (2) oneself as a learner of mathematics, (3) mathematics teaching, and (4) mathematics learning. As stated by himself, this framework is only based on a classification of published belief statements, and is not underpinned by any cognitive or meta-cognitive theory of learning. That is, he derived the four categories from existing belief statements in the literature. For example ‘beliefs about mathematics’ emerged from statements like *Mathematics is computation* and *Formal mathematics has little to do with real thinking*, which had been extensively exploited in earlier research (e.g. in [24], [84]). These categorisations, although useful for classifying existing outcomes, are disappointing from a theoretical standpoint because there is no further premise or theory beyond them. This led Pehkonen to the conclusion that his categories were inadequately defined; “they are actually artificial, in the sense that many beliefs belong to more than one of the four groups” ([80], p.14). For instance, some of his statements like “*Only geniuses are capable of discovering or creating mathematics”* and “*Mathematics is created only by very prodigious and creative people”* could indeed fall into both ‘beliefs about mathematics’ and ‘beliefs about oneself’ categories. In this light the acknowledgment of the epistemological belief theories of learning ([57], [85]), which include ‘innate ability’ and ‘omniscient authority’—corresponding to the two statements respectively—would have helped him to set his framework in a more authorised context.

More current studies (e.g. [86], [9]) follow similar patterns in the development of theoretical models, which lead to similar concerns. For instance De Corte’s model, based on an earlier review [10], is also disappointing from a theoretical view. His model put together several belief dimensions traditionally studied separately, but without considering appropriately the existing theories. This becomes evident when several of his designed items do not certainly capture the meaning of the constructs. For example, for evaluating the sub-dimension self-efficacy beliefs, De Corte ([9], p.12) uses items such as “*I can understand the course material*” and “*I expect to get good grades on maths test*” which do not specifically relate to self-efficacy but other related constructs. In fact, the former item relates to ‘self-confident’ and the latter relates to ‘expectation of success’. Importantly, although these labels share common ground they represent different constructs. ‘Mathematics self-efficacy’ refers to a situational or problem-specific assessment of an individual’s confidence in her/his ability to successfully perform a task [55]. ‘Self-confidence’ addresses a more generalised or global confidence in one’s ability to learn mathematics [65], and ‘expectation of success’ is normally related to the prediction of academic performance. Again, a more thorough review of the literature would have helped De Corte to put ‘self-efficacy’ in relation to other related constructs and to evaluate this dimension in a more precise way.

In contrast, the models proposed in educational psychology are typically well grounded in existing socio-cognitive or motivational theories. For example, the motivational beliefs framework proposed by Pintrich [87] emerged directly from a revision of the existing motivational theories rather than from a categorisation of single beliefs. This approach enabled Pintrich to precisely specify the proposed components, as well as to predict their relations with other relevant constructs.

### 4.2 Clearly defining the phenomenon under research

Some researchers have not only ignored existing theories but have failed to provide a general definition of the phenomenon to be measured. For example, Mason [88], in her validation of Kloosterman’s and Stage’s instrument [66], begins her paper with an extensive introduction about the important role that beliefs play in the learning of mathematics, but does not give or adopt any definition of beliefs or beliefs systems. According to De Vellis’ [14] guidelines on construct conceptualisation, it is important for researchers to put concepts like beliefs in context with respect to related concepts, such as ‘knowledge’ and ‘attitudes’. This is a common methodological shortcoming in mathematics education research, as it violates the most basic principles of investigation: how can a variable be accurately measured unless it is clearly stated?

Yet, given a general definition does not guarantee a sound conceptualisation. Several proposed definitions in mathematics education, used later for scale development purposes, are problematic as they do not clearly define the boundaries of the variable under study. For instance, Thompson’s ([2], p.132) definition, adopted by Lam [70] among others, considers conceptions to be “conscious or subconscious beliefs, concepts, meanings, rules, mental images, and preferences”. Hart ([89], p.44) describes beliefs as “certain types of judgment about a set of objects”, and Beswick ([90], p.41) refers to beliefs as “anything that an individual regards as true”. In fact, these definitions are meaningless regarding their practical application. Thompson defines conceptions in relation to other terms which she fails to define. Hart and Beswick’s definitions are so broad that they are unusable in a practical context. For example, is Beswick considering as part of ‘beliefs’ statements which are verifiable, in other words objective knowledge?

The use of such broad definitions hinders the scale design process because researchers tend to unconsciously use the same items to tap theoretically different phenomena. For instance, items like *Mathematics has many everyday uses* and *Mathematics is useful in people’s daily life* have been widely used to identify beliefs (e.g. [53], [67], [9]), attitudes (e.g. [91], [65]) and conceptions (e.g. [68], [92]) about the ‘usefulness of mathematics’. Similarly, statements such as *My teacher has not inspired me to study mathematics* have been used to study student beliefs (e.g. [53]), attitudes (e.g. [65]), conceptions (e.g. [8]) and views (e.g. [46]) about the teacher’s role. Similar citations can be found in constructs about ‘mathematics teaching’, ‘mathematics learning’, ‘mathematics as a subject’, ‘confidence in mathematics’, or ‘difficulty of mathematics’.

Importantly the use of broad definitions, which undermine the research on beliefs, is very common in current articles. Authors are not only creating but also adopting, from others, definitions that are not useful from a practical perspective. For example, Thompson’s [2] definition of conceptions, discussed above, has been frequently adopted by a number of authors over a long period—in 2015 alone it was quoted by Yang and Leu [59], Rolka and Roesken-Winter [93], and Sumpter [58] among others. Similarly, Schoenfeld [24] broad definition “beliefs are one’s mathematical worldview, the perspective one takes to approach mathematics and mathematics tasks”, has been adopted in the last few years by several researchers including Tsiapou [94], Jackson [95], and Bofah [60] to frame their studies. The above shows that this vague conceptualization of beliefs is a problem of the past that has continuity in the current literature.

### 4.3 Defining the specific sub-dimensions of the constructs

There are studies that provide a definition of the intended phenomena, but have failed to specifically define their sub-dimensions. Tapia and Marsh [96] defined ‘attitudes toward mathematics’ as multidimensional phenomena comprising constructs like ‘confidence’ and ‘enjoyment’, which they failed to describe. Although the authors quoted several sources in relation to these constructs, they did not define them in any specific way. This lack of conceptualisation was later reflected in a poor scale design. For instance, for tapping into the construct ‘confidence’, Tapia and Marsh used items like *Studying mathematics makes me feel nervous*, which has been used as an indicator for ‘anxiety’ [54]. Thus a suitable definition of the term ‘confidence’ and a revision of the related theories could have prevented the authors from employing items which do not capture the single meaning intended.

The case of Gil-Ignacio [97], who relied on McLeod’s [7] model, was similar. They listed several belief dimensions, which they unsuccessfully delineated for scale development purposes. For instance, they described ‘beliefs about oneself as a learner of mathematics’, as the “confidence and security in oneself, expectations of achievement, the desire for mastery of the subject, the social value provided by the subject, and the attributions of the causes of success to effort” (p.21). However, they failed to define each sub-construct, and as a consequence, a single scale was designed for capturing the meaning of all of them, instead of developing a separate scale for each. These sub-constructs have been clearly theorised and defined in educational psychology as separated entities, and scales have been proposed to evaluate them. ‘The desire for mastery of the subject’ for instance has been widely investigated under goal theories (e.g. [98], [99]), and several scales already exist for measuring different dimensions of goals (e.g. [100]). Similarly the ‘social value of the subject’ has been widely defined by motivational theories of learning (e.g. Eccles et al.’s [101] ‘expectancy-value theory’ or **‘**the self-determination theory’, [83]), and several measurements have been proposed to evaluate this variable, for example the Motivated Strategies for Learning Questionnaire (MSLQ) of Pintrich [56]. Gil-Ignacio. [97] may have drawn on this literature, and by not doing so have designed new scales which do not capture the intended meaning and which have brought more confusion to the field. Most recent papers show analogous shortcomings with similar consequences; see for instance Caballero [102] in their conceptualisation of ‘self-confidence’, Hannula [5] in their conceptualisation of ‘self-beliefs’, and Bofah [60] in their conceptualization of ‘self-concept’.

It is worth highlight that large-scale projects, that have conceptualised and refined the phenomena under research for several years, show also problems to clearly define the sub-dimensions of the constructs. For instance, TIMSS has been measuring student attitudes toward mathematics since 1995 [103], under specific dimensions such as ‘students like learning mathematics’, ‘students value mathematics’ and ‘students confident in mathematics’. Although these constructs have been refined, through subsequence studies in the last two decades (e.g. TIMSS 1995 [103], TIMSS 1999 [104], TIMSS 2015 [105], TIMSS 2017 [106]), and have been located within the existing motivational ([83], [107]) and self-related theories [108], their conceptualisations still remain ambiguous. For example, Mullis, [105] claim to measure ‘maths self-concept’ through ‘students confident in maths’ and more precisely through items like *I usually do well in maths*, *I am not good at maths*, *Maths makes me nervous*, and *Maths is harder to me than for many of my classmates* which tap meanings from well-differentiated constructs. In fact, the first of the aforementioned items refers to ‘self-perceived competence’ or ‘academic performance’ [109], the second one has been broadly used to tap ‘self-concept’ [110–113], the third one relates to ‘anxiety’ ([114], [115]), and the fourth one refers to a ‘self-perceived competence in relation to others’, focused on a social comparison [99]. Although the four belief items share a common ground, they measure different constructs, and thus the empirical results gained from this multi-item scale will certainly lead to misleading conclusions about the construct under evaluation (self-concept in this case). In the current situation, a more specific definition of ‘self-concept’, using for example the conceptualisation and instruments refined by Marsh (an authority in the evaluation of self-concept [110–113]), would have helped TIMSS to achieve more reliable outcomes.

### 4.4 Item pool design for a scale development purpose

Several studies provide a definition of the phenomena under research, and also clearly state their sub-dimensions; see for example the work of Fennema [65]. Kloosterman and Stage [66], Op’t Eynde [10], Op’t Eynde [67], OECD [116]). However, these studies often fail to use appropriated statements (or items) to exemplify and measure the stated sub-dimensions by a scale development process.

For example, the Programme for International Student Assessment (PISA) [116] seems to have thoroughly located self-belief constructs, such as ‘self-efficacy’, ‘self-concept’ and ‘anxiety’, within the existing related theories to specifically define such constructs. PISA used, for instance, Bandura’s Social Learning Theory to appropriately define ‘self-efficacy’ [117] as “students’ convictions that they can successfully perform a given academic tasks” (e.g. *I can do the most difficult problem in maths* [118]). In a similar vein, the subsequent Marsh studies: [110–113] were employed to theorise and conceptualise ‘self-concept’ as “one belief in their own abilities […] e.g. *I am not good at maths*” ([119], p.87). A similar approach was employed to adequately define ‘anxiety’ and other related constructs. Locating the constructs within the appropriated theories and delivering explicit definitions and examples, as the above stated, helped PISA to provide accurate indicators and ensuring *content validity*. The studies of Fennema and Sherman [65], Kloosterman and Stage [66], and Op’t Eynde et al. [10] have carried a similar approach to that of PISA for conceptualising other belief constructs.

The only criticism that can be made to the above-mentioned studies relates to the design of the scales used to measure the constructs. These scales sometimes lack of internal and cross-cultural validity. It is for example common to observe double-barreled items with two or more ideas linked, undermining internal validity. For example the following items

- *In addition to getting a right answer in maths*, *it is important to understand why the answer is correct*;
- *Mathematics makes me feel uncomfortable*, *restless*, *irritable*, *or impatient*.
- *Our teacher gives us time to really explore new problems and to try out possible solution* strategies; or
- Sharing *ideas and verifying conjectures is an important part of doing mathematics*,

have been extensively used in well-known scales like those of Kloosterman [66], PISA 2013 [120], Op’t Eynde [67], and Taylor [121]. These items are ambiguous and lead to poor measurements as they allow for or even encourage different interpretations. The last item contains for example two different ideas–*sharing ideas* and *verifying conjectures—*which in fact relate to different levels of cognitive demand. How can these items be coherently answered in a scale response format if they address different constructs? These items violate the most basic principle of scale development which states that *scales should aim to assess a unique meaning* ([122], [123], [124]). Although scales, as well as constructs, can be developed in a relatively broad or narrow way depending on the research necessities, they should include only one idea [14]. This widespread use of double-barreled items may be due to the fact that researchers in mathematics education have often used statements from earlier exploratory studies to design measurement scales, without considering the scale validity techniques employed by psychometricians. In other words, inadequate items for a scale development purposes seems to have been used in mathematics education. Using scale validity techniques would have avoided the use of problematic items. It is also common practice to use lengthy and negatively-orientated items such as *I see mathematics as something I will not use very often when I get out of high school* (Doepken [125]) which also diminishes clarity and can confuse the respondent.

Several scales in the literature have also being criticised for their lack of cross-cultural validity. For example, expressions commonplace in, say, self-efficacy scales (e.g. those of Fennema [65], PISA 2012 [119], and Op’t Eynde [126], Ren [61]) like *I am confident that*…, *I am sure that*…, and *I am certain that*…, proved problematic with respect to Spanish equivalents, no least because confident, sure and certain imply subtly increasing levels of certain in English, which cannot be accommodated in Spanish [127]. “The Spanish translation of *I am confident that* is *estar seguro de que*. However, this back-translates as *I am certain that* or *I am sure that*, missing the uncertainty implicit in the original. *I am confident of doing something* translates as *confío en hacer algo*. However, confío alludes to an implicit sense of hope, which is lacking in the English confident. Furthermore, *I am certain that* translates as *estoy seguro*, which back-translates as I am sure that. Such matters highlight the difficulty that the expressions typically employed in self-efficacy scales have for establishing conceptual and linguistic equivalence” ([127], p.554).

The above analysis of the literature shows that the mathematics research community has often failed to provide sound conceptualisations of beliefs, and has overlooked existing theories and definitions related to the constructs. Several studies have also failed to design belief scales, violating basic principles of the scale development process like internal and conceptual validity. The incorrect design of these scales has led to wrong conclusions about the phenomena under research.

## 5. Conclusions

This article shows that the field of student mathematics-related beliefs requires a more systematic conceptualisation and measurement of the phenomenon under research. It has been shown there is a clear disagreement on the definition and categorisation of beliefs. After an analysis of the most relevant journal articles, conference proceedings, large-scale projects, and widely used instruments in the literature, this study shows that authors often fail in at least four issues related to construct conceptualisation and measurement. Firstly, existing theories related to the constructs are normally not considered. This is an important issue—if the nature of the construct under evaluation is unclear, a suitable conceptualisation cannot be formulated [14]. Secondly, definitions are often too broad failing to confine the construct under research. This creates overlap with other related constructs, leading to problems when classifying beliefs and hampering appropriate distinctions. Thirdly, the existing belief sub-dimensions are rarely defined and thus not explicitly distinguished. The use of general or unbounded conceptualisations often leads researchers to treat different categories of beliefs as they were conceptually the same or vice-versa. Fourthly, the multi-item scales used to measure the constructs under research do not always meet the necessary validity-related criteria. The use of scales that do not ensure, for instance, internal and conceptual validity will not provide reliable results about the variable under evaluation ([74], [79]). This in turn will affect a posterior re-conceptualisation of the own construct [13].

It is thus important that future studies consider existing theories to delimitate the variable under conceptualisation. Narrowing definitions down is essential, as it is important providing clear examples of what these definitions do and do not encompass. It is also necessary to provide explicit definitions for the emerging sub-categories of beliefs. Likewise, the validation of the constructs by means of scale development, and testing, will help to provide further consistency to the conceptualisations, and facilitating accurate re-conceptualisations. Accurate conceptualisations and measurements will not only facilitate communication between researchers, allowing results comparison and a better understanding of beliefs, but also more reliable analyses and thus more accurate results about the significance of beliefs.

There is thus an urgent need for a re-conceptualisation of the field of student mathematics-related beliefs by means of a comprehensive model, which take the above issues into consideration. This model should be achieved through an interactive process of conceptualisation and measurement, until a sound conceptualisation is reached. Thus, iterative studies in which constructs are conceptualised and re-conceptualised according to empirical analyses are necessary. This iterative process will not only help to determine and define the phenomenon under scrutiny, but also to provide further consistency and reliability, because construct consistency is achieved when the same results are obtained under fairly similar conditions ([75], [14]).

We belief that the fundamental problem, preventing the conceptualisation of such comprehensive model, is that researchers in mathematics education have not used the methodological tools necessary to measure elusive phenomena like beliefs. Although, in the last few years, some authors in the field of student mathematics-related beliefs have employed in their studies sophisticated tools from psychometricians (e.g. confirmatory factor analysis or structural equation modelling), there is still a necessity for a more methodical analysis of this variable. We think that drawing on the psychometricians theory, and learning from those who are experts in conceptualising and measuring elusive phenomena, will help to clarify our field of research. Otherwise, the field of the student mathematics-related beliefs is in danger of isolating itself from others, often more knowledgeable and experienced, working on what we might call the mother fields.

## Supporting information

S1 File Dataset. (XLSX)
